# KDELR2 Competes with Measles Virus Envelope Proteins for Cellular Chaperones Reducing Their Chaperone-Mediated Cell Surface Transport

**DOI:** 10.3390/v11010027

**Published:** 2019-01-04

**Authors:** Vishakha Tiwarekar, Markus Fehrholz, Jürgen Schneider-Schaulies

**Affiliations:** Institute for Virology and Immunobiology, University of Würzburg, 97078 Würzburg, Germany; Vishu7yende@gmail.com (V.T.); m.fehrholz@monasteriumlab.com (M.F.)

**Keywords:** measles virus, KDELR2, calnexin, GRP78, surface transport

## Abstract

Recently, we found that the cytidine deaminase APOBEC3G (A3G) inhibits measles (MV) replication. Using a microarray, we identified differential regulation of several host genes upon ectopic expression of A3G. One of the up-regulated genes, the endoplasmic reticulum (ER) protein retention receptor KDELR2, reduced MV replication ~5 fold when it was over-expressed individually in Vero and CEM-SS T cells. Silencing of KDELR2 in A3G-expressing Vero cells abrogated the antiviral activity induced by A3G, confirming its role as an A3G-regulated antiviral host factor. Recognition of the KDEL (Lys-Asp-Glu-Leu) motif by KDEL receptors initiates the retrograde transport of soluble proteins that have escaped the ER and play an important role in ER quality control. Although KDELR2 over-expression reduced MV titers in cell cultures, we observed no interaction between KDELR2 and the MV hemagglutinin (H) protein. Instead, KDELR2 retained chaperones in the ER, which are required for the correct folding and transport of the MV envelope glycoproteins H and fusion protein (F) to the cell surface. Our data indicate that KDELR2 competes with MV envelope proteins for binding to calnexin and GRP78/Bip, and that this interaction limits the availability of the chaperones for MV proteins, causing the reduction of virus spread and titers.

## 1. Introduction

Measles virus (MV) replication is reduced by more than 90% in ectopically A3G expressing Vero cells [1]. We recently described that one of the A3G regulated host cell factors, REDD1 (regulated in development and DNA damage response-1), affects MV replication via regulation of mTORC1 [2]. Another host cell factor, KDELR2, was significantly increased upon the ectopic expression of A3G [2], requiring closer investigation. The KDELR family of receptors plays an important role in the retrieval of ER resident chaperones containing the C-terminal tetra peptide sequence KDEL (Lys-Asp-Glu-Leu) [3]. Activation of the seven-transmembrane KDEL receptors results in the activation of src family kinases coordinating the secretory pathway [4]. By this mechanism, chaperones regulate the maturation and fate of proteins to prevent excessive aggregation in the ER-Golgi network. KDELR receptors are primarily localized in the Golgi where they sort escaped chaperones to COPI vesicles and initiate their retrograde transport. Ligand binding or over-expression results in the redistribution of KDEL receptors to the ER [5,6]. Binding of ligands to KDELR2 is a pH dependent process, which is efficient at acidic pH and weaker at basic pH [7]. Therefore, the binding of chaperones to KDELR2 in the acidic Golgi environment is strong, and after transport to the neutral ER, the cargo is released in the ER [8]. Although already identified in 1990, the complete functions of KDELR family proteins are poorly understood. Recent findings have suggested that KDELR-ligand binding can induce signaling cascades regulating cellular secretory traffic [9], growth, differentiation, and immune responses [10]. These findings have shown that KDEL receptors exert their effects at multiple levels serving additional unidentified novel roles.

For maturation and viral egress, viral proteins must traffic across the ER and the Golgi to reach their assembly sites at the plasma membrane, which is assisted by various ER proteins (reviewed in [11]). KDELR mediated pH dependent transport across these compartments is exploited by various viruses. For example, the dengue virus 1 (DENV1) prM protein interacts directly with KDELR1 and KDELR2, which supports transport of the viral envelope proteins to the plasma membrane [12]. JEV (Japanese encephalitis virus), another member of the Flavivirus family, is also dependent on KDELR1 mediated viral particle transport to the plasma membrane [13]. In the case of the vaccinia virus, host cellular membranes are delivered to virions by KDELRs and coatomer proteins [14]. Interestingly, FIPV (feline infectious peritonitis coronavirus) glycoproteins possess the ER retention signal KTEL to delay their release from the ER, which ensures the correct maturation of these proteins [15]. Various other viruses such as HCV (hepatitis C virus) are known to extensively use ER membrane webs for particle assembly [16].

No interaction of MV proteins with KDELRs has been reported in the literature. Interestingly, the ER chaperones GRP78/BiP, calreticulin, and calnexin have been shown to play an important role in the maturation and surface expression of MV-H in infected cells [17]. Furthermore, after the infection of cells with MV or canine distemper virus (CDV), or by ectopic viral glycoprotein expression, ER stress is induced and the expression of various chaperones is enhanced [17,18].

Here, we show that KDELR2 levels were increased in A3G expressing Vero cells. KDELR2 over-expression in Vero and CEM-SS T cells reduced the MV titer significantly. Our data suggest that KDELR2 competes with MV-H for binding to various chaperones, and that this is responsible for the reduced expression of MV-H on the cell surface, reduced viral syncytium formation, and reduced viral titers.

## 2. Materials and Methods

### 2.1. Cells and Viruses

African green monkey kidney Vero cells (ATCC CCL-81) were transduced with the following vectors to generate transduced Vero cells: Vero-023 with the empty vector pCMS28, Vero-KDELR2 with pF6gW expressing human KDELR2, Vero-A3G with pCMS28-A3G (same as in [1]), and Vero-KDELR2sh2 with pF6gW expressing KDELR2-specific shRNA (see below). Transduced cells were grown under selection pressure with 5 µg/mL of puromycin [19] or FACS sorted based on DsRed2 fluorescence. Human embryonic kidney 293T cells and Vero cells were cultivated in DMEM medium containing 5% FCS, 100 U/mL penicillin/streptomycin, and non-essential amino acids.

The attenuated vaccine strain MV-Edmonston and the recombinant Edmonston-based MV strain rMV-eGFP expressing eGFP [20] were propagated using Vero cells. To assess the effect of individual genes on viral replication, Vero cells transduced with respective vectors were seeded in 6-well plates and infected at a MOI of 0.1. The virus was harvested after the indicated times by freezing and thawing the complete culture, thus the cell associated and supernatant virus was harvested together.

### 2.2. Cloning of Lentiviral Expression Plasmids and Production of Pseudotyped Particles

For shRNA expression, we used the vector F6gW-DsRed also expressing DsRed2 as a control for transduction efficiency as described in [21]. To clone the shRNA expressing vectors, we selected siRNA sequences from published mRNA sequences by the program provided by Block-iT RNAi designer (Invitrogen, Carlsbad, CA, USA) and used DNA oligonucleotides for cloning into pF6gW-DsRed (all shRNA sequences can be provided on request). The oligonucleotides were aligned and cloned into the HpaI and XhoI sites of F6gW-DsRed. The sequences of the clones were confirmed by sequencing. The selected KDELR2-specific shRNA expression construct 2 (KDELR2sh2) most efficiently silenced the expression of KDELR2 and was used for further experiments. Lentiviral expression vectors for KDELR-Flag were generated using the BamH1-SacII fragment of KDELR2-expression plasmid RC200007 (OriGene) and cloned into the BamH1-EcoR1 sites of F6gW. The sequences of the cloned plasmids were confirmed by sequencing.

VSV-G-pseudotyped viral particles were produced by the transfection of HEK-293T cells with plasmids pVSV-G, pRSVrev, and pMDLg/pRRE and pF6gW-based retroviral vectors using polyethylenimine (PEI; 25 K; Polysciences Inc.; Warrington, PA, USA). Two days after transfection, the supernatant was harvested and filter sterilized (0.4 µm). The particle preparations were concentrated using Amicon^®^ Ultra 100K centrifugal filter units (Millipore, Burlington, MA, USA) or PEG-it (System Biosciences, Palo Alto, CA, USA) and titrated. Vero cells were transduced with these pseudotyped lentiviral particles at a MOI of 30.

### 2.3. Antibodies and Flow Cytometry

In immunoblotting, we used the rabbit anti-KDELR2 (Sigma SAB1401554, St. Louis, MO, USA), rabbit anti-GAPDH (Santacruz sc-25778; Dallas, TX, USA), rabbit anti-GRP78 (Pierce, PA5-11418, Hubei, China), and rabbit polyclonal anti MV-H cytoplasmic domain (Eurogentec, Liège, Belgium). For flow cytometric analysis and immunofluorescence, rabbit anti-calnexin (Pierce, PA1-30197), rabbit anti-GRP78 (Pierce, PA5-11418, Hubei, China), rabbit anti-calreticulin (Pierce PA3900, Hubei, China), mouse monoclonal anti-FLAG (Sigma, F1804, St. Louis, MO, USA), mouse monoclonal anti MV-H L77 [22], mouse monoclonal anti MV-F A504 [22], Alexa 488 anti-mouse (Life technology A11001, Carlsbad, CA, USA), Alexa 594 anti-rabbit (Life technology, A11012, Carlsbad, CA, USA), and anti-mouse APC (Biolegend, 406414, San Diego, CA, USA) were used.

For flow cytometry, 1 × 10^5^ cells were infected with MV-eGFP at a MOI of 0.1. After 2 h, the virus inoculum was replaced by medium containing 200 µM FIP (Fusion Inhibitory Peptide; Bachem H9430, Bubendorf, Switzerland) throughout the incubation period to facilitate single cell analysis by preventing syncytium formation. For cell surface expression analysis, cells were stained with respective antibodies at 4 °C for 30 min to 1 h. For total expression analysis, cells were fixed and permeabilized with 4% paraformaldehyde and 0.1% Triton X-100 in FACS buffer (PBS containing 0.4% BSA and 0.02% sodium azide). Cells were resuspended in FACS buffer and data were acquired using a LSR II flow cytometer (BD) and the data were evaluated using FlowJo (BD, Franklin Lakes, NJ, USA) software.

### 2.4. Immunofluorescence Staining

Control and KDELR2 expressing Vero cells (2 × 10^4^) were seeded on a Nunc™ Lab-Tek™ II Chamber Slide and infected a day later with recombinant MV at a MOI of 1 for 2 h. The viral inoculum was then replaced with media containing FIP throughout the incubation period. Twenty-four hours after infection, cells were fixed in 4% paraformaldehyde and then permeabilized in 0.1% Triton X-100 for 15 min at 4 °C. The cells were blocked in 5% bovine serum albumin fraction V (Serva 11930.2, Rosenheim, Germany) at room temperature for 2 h. Primary antibodies were diluted in PBS containing 1% FBS and incubated overnight at 4 °C. The MV-H protein was stained using monoclonal mouse anti MV-H L77 (1:100). Cells were then washed three times and stained with anti-mouse Alexa 594 (1:400) for 1 h at 4 °C, and blocked for 1 h with 10% normal mouse serum (Jackson ImmunoResearch 015-000-001, West Grove, PA, USA) before staining for KDELR2-Flag using mouse anti-Flag Alexa 488 (1:100) for 1 h at 4 °C. The nuclear staining was done using DAPI (1:1000), then slides were mounted using Fluoromount-G (SouthernBiotech, 0100-01, Gelderland, Netherlands). The images were captured using LSM 780 (Zeiss, Oberkochen, Germany) and processed using Zen2012 software (Oberkochen, Germany).

### 2.5. Immunoblotting and Co-Immunoprecipitation

Cells (5 × 10^6^) were lysed at 4 °C for 1 h in 1 mL of lysis buffer (50 mM Tris-HCl, pH 8.0, 150 mM sodium chloride (NaCl), 1.0% Igepal CA-630 (NP-40), 0.5% sodium deoxycholate, 0.1% sodium dodecyl sulfate (SDS)) containing a complete protease inhibitor cocktail (Sigma, P8340, St. Louis, MO, USA) and 1 mM DL-Dithiothreitol (DTT). The protein quantification was done using the bicinchoninic acid (BCA) assay. An equal amount of proteins was heated at 95 °C for 5 min in reducing Laemmli sample buffer (50 mM Tris HCl pH 6.8, 2% SDS, 10% glycerol, 1% β-mercaptoethanol, 12.5 mM EDTA, 0.02% bromophenol blue) and applied to 10% SDS PAGE. For co-immunoprecipitation, mock-treated and MV infected Vero cells (023 and KDELR2 expressing cells) were lysed at 4 °C for 1 h with 1 mL of IP lysis buffer (50 mM Tris-HCl pH 7.5, 150 mM NaCl, 50 mM NaF, 2 mM EDTA, 0.5% sodium deoxycholate, 1% NP-40) containing 10 µL of protease inhibitors. Cell debris was removed by centrifugation at 13,000 rpm for 5 min at 4 °C. Lysates were then incubated with pre-washed mouse anti-Flag M2 affinity gel (Sigma, St. Louis, MO, USA) for 2 h at 4 °C. Beads were then washed three times in IP buffer and three times in PBS. Bound proteins were eluted by boiling in reducing protein sample buffer for 5 min. The bound proteins were eluted by heating beads in reducing Laemmli sample buffer at 95 °C for 5 min and then applied to 10% SDS–PAGE. Proteins were blotted semidry on nitrocellulose membranes (Amersham, GE Healthcare Bio-Sciences, Pittsburgh, PA, USA) followed by blocking with 10% dry milk (AppliChem, Cinisello Balsamo, Italy) in PBS or 5% BSA in the case of KDELR containing 0.05% Tween-20. The membranes were then incubated with specific primary antibodies and anti-mouse (Cell Signaling, #7076S, Danvers, MA, USA) or anti-rabbit (Cell Signaling, #7074S, Danvers, MA, USA) HRP-conjugated secondary antibodies. Signals were visualized using a chemiluminescent FemtoMax™ super sensitive HRP substrate (Rockland, NY, USA).

### 2.6. Real Time qPCR

The total RNA was isolated independently three times from control Vero 023 and Vero A3G cells using the GeneElute Mammalian Total RNA kit (Sigma) as per the manufacturer’s instructions. Isolated RNA was reverse transcribed in cDNA using the RevertAid first strand cDNA synthesis kit (Fermentas). Gene specific primers were used to amplify the target genes. Real time PCRs were performed using the 2X SYBR Green qPCR Master mix (Bimake, Houston, TX, USA ) and amplified on the LightCycler 2.0 real-time PCR system (Roche, Basel, Switzerland). Primers for KDELR2 were forward: 5′ CTCTTCCTCTGCTGCGAAGT 3′ and reverse: 5′ ATGGAAAGCAGCCAAAACTC 3′. Cycling conditions were as follows: 95 °C for 180 s, followed by 45 cycles of 95 °C 10 s, 60 °C for 30 s, followed by 95 °C for 10 s, 60 °C for 60 s, 95 °C for 1 s, followed by 37 °C for 30 s. A melting curve analysis was performed after each run to verify single PCR products. The relative copy number was calculated using the ∆∆Ct method.

### 2.7. Statistical Analysis

Statistical analysis was performed using GraphPad Prism 6 (GraphPad, San Diego, CA, USA). Two groups were analyzed using the unpaired two-tailed Student’s t-test and more than two groups were analyzed with one-way ANOVA. P-values lower than or equal to 0.05 were considered statistically significant (* = *p* < 0.05, ** = *p* < 0.01, *** = *p* < 0.001). The data represent the mean ± SD of at least three independent experiments.

## 3. Results

### 3.1. KDELR2 Over-Expression in Vero and CEM-SS T Cells Reduces MV Titers

**Figure 1:**
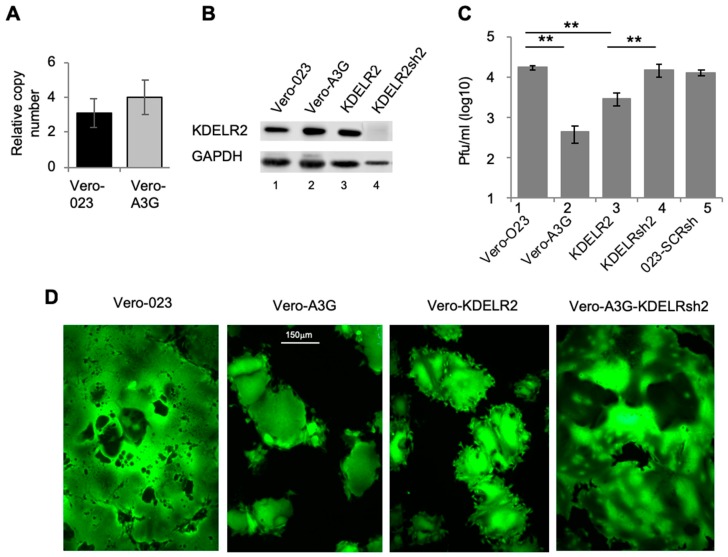
The A3G upregulated gene KDELR2 reduces MV replication in Vero cells. (**A**) Total RNA from Vero 023 and Vero A3G was isolated and reverse transcribed into cDNA. KDELR2-specific cDNA was then amplified using SYBR-Green Real-Time qPCR (*n* = 3). (**B**) The protein expression of KDELR2 was analyzed using Western blot. Equal amounts of cell lysates were separated on 12% SDS-PAGE and transferred on a nitrocellulose (NC) membrane. Target proteins were probed with primary KDELR2 antibody and HRP conjugated secondary antibody then developed using ECL (lane 1: Vero 023, lane 2: Vero A3G, lane 3: Vero KDELR2, lane 4: Vero A3G + KDELR2shRNA). (**C**) Transduced Vero cells were infected with MV eGFP at MOI of 0.1. The titer of newly synthesized virus in these cells was determined 48 h post infection on Vero cells (*n* = 3). Significance was calculated using the Student’s t test (** *p* < 0.01). (**D**) Representative micrograph of MV syncytium based on eGFP fluorescence 72 h post infection (magnification × 100, size bar: 150 µm).

Real time qPCR showed a tendency of increased KDELR2 mRNA expression in Vero-A3G cells when compared to Vero-023 cells (empty vector control) (Figure 1A). Accordingly, the protein expression of KDELR2 was increased in Vero-A3G cells 1.3-fold [2] (Figure 1B, lane 2). To investigate the potential role of KDELR2 in the inhibition of MV replication, Vero cells were transduced with a KDELR2 expressing lentiviral vector. The KDELR2 protein expression in these Vero-KDELR2 cells was confirmed by Western blotting (Figure 1B, lane 3). Vero-KDELR2 cells, Vero-A3G, and Vero 023 cells were infected with rMV-eGFP and the titers of newly synthesized MV were determined after 1, 2, and 3 days (Figure 1C). Ectopic expression of KDELR2 reduced the MV titer by approximately 88% (Figure 1C, lanes 3) in comparison to 97.7% in the case of A3G (Figure 1C, lanes 2). To confirm the role of KDELR2 in the A3G mediated inhibition of MV, KDELR2 levels were depleted in Vero-A3G cells using KDELR2-shRNA (Figure 1B, lane 4). As the antiviral effect in Vero-A3G cells is not only due to KDELR2, but other effectors are also involved such as REDD1 [2], we expected a partial abrogation of the antiviral effect by KDELR2-specific shRNA. Interestingly, the silencing of KDELR2 in Vero A3G cells significantly increased the viral titer to a level comparable to that found in Vero-023 control cells (Figure 1C). This is probably due to the fact that shRNA treated cells express less KDELR2 than Vero-023 cells, thus the antiviral effect exerted by KDELR2 in Vero-A3G cells was over-compensated. The syncytium formation observed in these various cell lines reflected the findings with the viral titers (Figure 1D).

**Figure 2:**
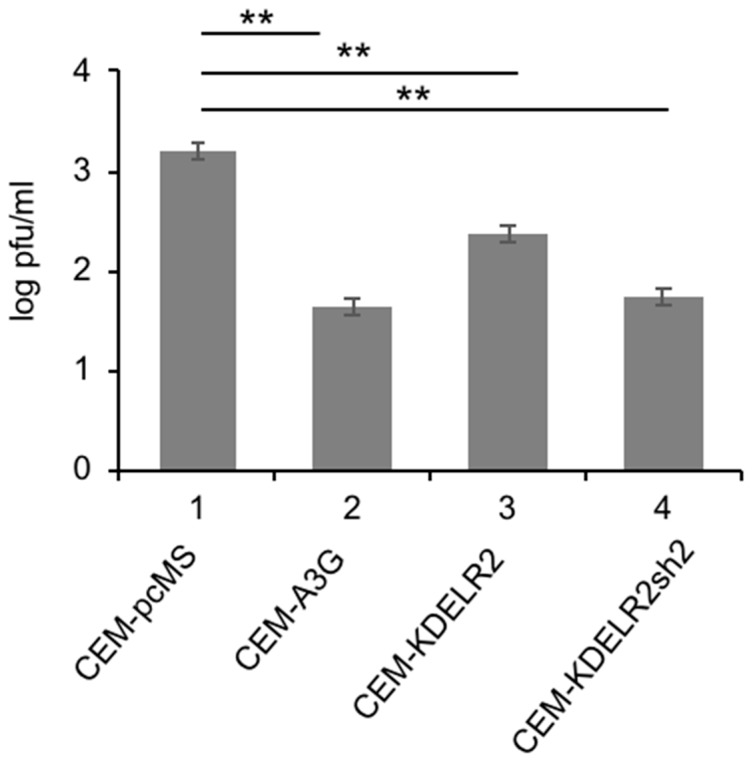
KDELR2 reduces MV replication in CEM-SS T cells. Transduced CEM-SS T cells were infected with MV-eGFP at a MOI of 0.1. The titer of newly synthesized virus in these cells was determined 48 h post infection on Vero cells (*n* = 3). Significance was calculated using the Student’s t test (** *p* < 0.01).

The primary target cells of MV are human CD150-positive lymphoid cells, whereas the transduced Vero cells are non-human epithelial cells with defects in the IFN system, and thus not optimal for the investigation of host factors. Therefore, we assessed the effect of KDELR2 on MV replication in the human T cell line CEM-SS. As found in Vero cells, MV titers were significantly reduced in CEM-SS cells expressing A3G when compared to CEM-SS transduced with empty vector control pcMS (Figure 2). Investigation of the CEM-SS T cells expressing KDELR2 also showed a significant reduction in MV titers. Interestingly, KDELR2-specific shRNA expression reduced the MV titer further (Figure 2, lane 4). This may be due to the fact that KDEL receptors may also be required for the proliferation of T cells, as has been shown for KDELR1, another member of the KDELR family [23]. These results indicate that too much KDELR2 as well as too little KDELR2 expression is sub-optimal for MV replication in this T cell line.

### 3.2. KDELR2 Reduces MV-H Surface Expression

**Figure 3:**
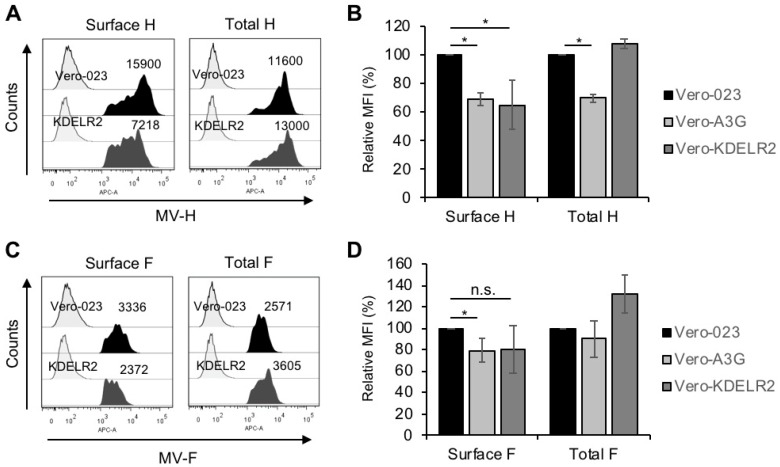
KDELR2 over-expression leads to a reduction of MV-H and -F surface expression. Vero-023, Vero-A3G, and KDELR2 over-expressing Vero cells (KDELR2) were infected with MV-eGFP at a MOI of 0.1 for 48 h. Cells were fixed and stained (surface expression), or fixed, permeabilized, and stained (total expression) with monoclonal antibodies to MV-H (**A**,**B**) and MV-F (**C**,**D**) and secondary antibodies. The MV-H and –F expression was analyzed by flow cytometry. Examples of representative experiments with isotype controls (left signals) and surface and total H (**A**) and F (**C**) expression (dark signals) are shown. The results of three experiments are summarized in bar graphs (**B**,**D**). Data of mean fluorescence intensities (MFI) were presented as a percent of control normalized to values of Vero-023 cells. Significances were determined using the Student’s t-test (* *p* < 0.05; n.s. = not significant).

Being involved in the transport of proteins between Golgi and ER, KDELR2 might eventually interact with the MV envelope glycoproteins H and/or F, which are synthesized and assembled in the ER [24]. Therefore, we quantified the cell surface and total expression of MV-H and -F in MV-infected Vero-023, Vero-A3G, and KDELR2 over-expressing cells on a single cell level by flow cytometry (FACS). The MV-H surface expression was considerably reduced by 35% and 32% on Vero-A3G and KDELR2 expressing cells, respectively, in comparison to the infected Vero-023 cells. MV-F expression was reduced by approximately 20% on the Vero-A3G cells and in the case of Vero-KDELR2 cells, there was a tendency of reduction (Figure 3). In contrast, the total MV-H expression was reduced by A3G, but not by KDELR2. Total MV-F expression was even increased in KDELR2 over-expressing cells (Figure 3). Thus, KDELR2 as well as A3G over-expression led to a reduction of the transport of MV envelope glycoproteins to the cell surface.

### 3.3. KDELR2 Does Not Interact with MV-H

**Figure 4:**
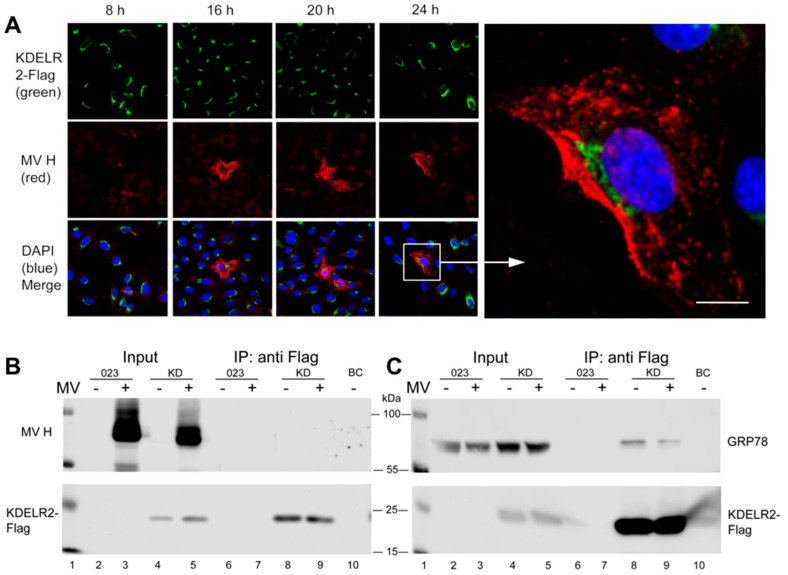
No detectable interaction was seen between KDELR2 and MV-H, but of KDELR2 with GRP78. (**A**) Transduced Vero cells over-expressing the KDELR2 (Vero-KDELR2-Flag) were infected with MV (MV-Edmonston not expressing eGFP) at a MOI of 0.1. After 8, 16, 20, and 24 h, cells were fixed and stained with antibodies to Flag and Alexa-488-conjugated secondary antibodies (green), to MV-H and Alexa-594-conjugated secondary antibodies (red), and DAPI (blue). Photomicrographs were taken using a confocal microscope (enlargement 400×; size bar = 20 µm). For co-immunoprecipitations, lysates of MV-infected and uninfected (as indicated) Vero-023 and Vero-KDELR2-Flag (KD) cells remained untreated (input, lanes 2 to 5) or were treated with anti-Flag antibody-conjugated beads for precipitation (IP: anti-Flag, lanes 6 to 9). Control precipitation was with unconjugated beads (BC, lane 10). Proteins were size separated by 10% SDS-PAGE, blotted onto nitrocellulose, and in (**B**) detected with antibodies to MV-H (polyclonal rabbit H45, upper panel), or to Flag detecting KDELR2 and secondary antibodies as indicated (lower panel). In (**C**), Western blots were treated with antibodies to GRP78 (upper panel), or to Flag detecting KDELR2 and secondary antibodies as indicated (lower panel).

In order to analyze this effect further, we tested if MV-H—although not containing the cognate KDEL sequence—may interact with KDELR2. This was investigated microscopically (Figure 4A) and by co-immunoprecipitation (Figure 4B). Microscopically, the signals for KDELR2 and MV-H were clearly separated and did not reveal any co-localization at all time points after infection, but rather excluded each other (Figure 4A). Co-immunoprecipitation studies were consistent with the lack of interaction of KDELR2 with MV-H (Figure 4B). These findings suggest that there is no direct interaction between KDELR2 and the MV-H.

### 3.4. KDELR2 Reduces Surface Expression of Chaperones

It has been reported previously that the chaperons calnexin, GRP78/Bip and calreticulin interact with MV envelope glycoproteins and support their transport to the cell surface [17], and that GRP78 and calreticulin contain KDEL sequences to interact with KDEL receptors [25]. We, therefore, investigated if the chaperones may mediate the KDELR2 effect on MV-H. First, we confirmed the KDELR2 interaction with GRP78 by immunoprecipitation. GRP78 was clearly co-precipitated with KDELR2 (Figure 4C, lane 8), and MV infection reduced this interaction (Figure 4C, lane 9) indicating that MV-proteins compete with GRP78 for interaction with KDELR2.

**Figure 5:**
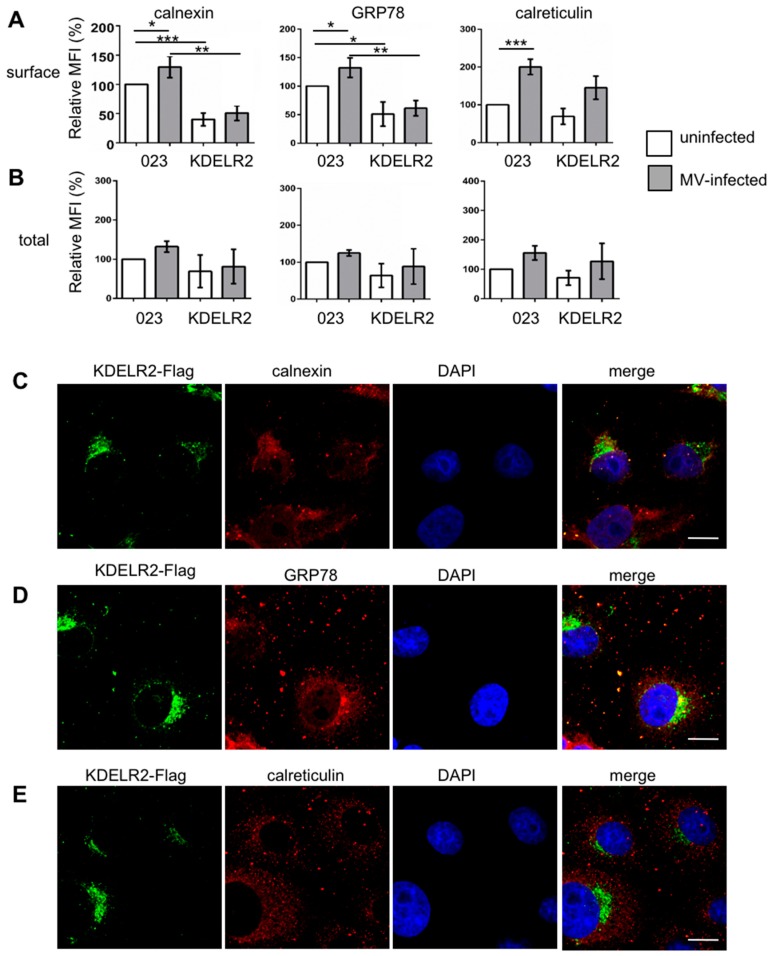
KDELR2 over-expression reduces the surface expression of calnexin and GRP78. Surface (**A**) and total (**B**) chaperone expression were quantified by flow cytometry. Vero-023 and KDELR2 over-expressing Vero cells (KDELR2) remained uninfected (open columns) or were infected with MV-eGFP for 48 h at a MOI of 0.1 (grey columns). Cells were stained after fixation (surface, **A**), and after fixation and permeabilization (total, **B**) with antibodies to calnexin, GRP78, and calreticulin as indicated, and the fluorescence intensities were evaluated by flow cytometry. Exclusively, eGFP-positive cells were evaluated as infected cells and eGFP-negative cells as uninfected cells. Data representative of three independent experiments were normalized to values of uninfected Vero-023 cells, and are presented as relative mean fluorescent intensities (MFI). Significances were determined using the Student’s t-test. The subcellular localization of the chaperones calnexin (**C**), GRP78 (**D**), and calreticulin (**E**) in comparison to KDELR2-Flag and nuclei (DAPI) was analyzed using a confocal microscope (enlargement 1000×; size bar = 20 µm).

Then, the surface and total expression of the chaperones calnexin, GRP78, and calreticulin was analyzed by flow cytometry in the control and KDELR2 over-expressing cells in the presence and absence of MV infection. KDELR2 over-expression significantly reduced the calnexin and GRP78 surface expression in uninfected and infected cells (Figure 5A). In contrast, quantification of the total expression of the chaperones revealed only a tendency of reduced expression in response to KDELR2 over-expression, which, however, was not significant (Figure 5B). Thus, the calnexin and GRP78 surface expression correlated with MV-H expression as determined above (Figure 3A), where both the chaperon and the MV-H surface expression were reduced by KDELR2 over-expression. The interaction of KDELR2 with calnexin and GRP78 was confirmed by co-localization studies. The chaperones co-localized with the KDELR2 (Figure 5C–E), with co-localization coefficients of 0.66 for calnexin, 0.75 for GRP78, and 0.54 for calreticulin (Pearson’s correlation coefficient; software ImageJ). These findings are in accordance with the model that MV envelope proteins require the chaperones calnexin and GRP78 for transportation to the cell surface, and that KDELR2 over-expression retains these chaperones in the ER, reducing the interaction of the chaperones with MV-H and its subsequent surface transport.

## 4. Discussion

We recently described several host genes which were differentially expressed upon ectopic expression of A3G, and identified REDD1 as one host factor mediating antiviral activity via regulation of mTORC1 [2]. Here, we further investigated the basis of the antiviral activity of A3G against MV in Vero and CEM-SS T cells. We found that the host factor KDELR2—by binding to and reducing the surface transport of chaperones—reduced the viral glycoprotein transport to the cell surface, viral spread via syncytium formation, and production of infectious viral particles. The data suggest that MV budding at the plasma membrane is impaired by this lack of MV glycoproteins.

The receptors of the four C-terminal amino acids KDEL are located in the ER-Golgi intermediate compartment and cis-Golgi, where they catch KDEL-bearing proteins escaping from the ER and re-transport them to the ER lumen. In contrast to our findings with MV, KDEL receptors have been described to support the replication of dengue [12], Japanese encephalitis [13], and vaccinia virus [14]. In the case of dengue virus, this is probably due to the fact that the viral prM protein is a direct target of KDELR and that nascent dengue viruses bud into the lumen of the ER and are translocated to the Golgi [12]. In the trans-Golgi network, prM is cleaved by cellular proteases and infectious dengue viruses are formed there. For the Japanese encephalitis virus, similar mechanisms may occur. In the case of the vaccinia virus, the interaction of KDELR with COPI and coatomer was shown to support the formation of infectious viral particles [14].

Interestingly, KDEL receptors as well as the chaperones GRP78, calreticulin, and calnexin are up-regulated by cells under stress conditions and viral infections as part of the stress response [17,18,26]. GRP78 binds the ER stress sensors PERK, IRE1, and ATF6 in their inactive forms, from which it is released upon ER stress, resulting in the activation of the associated signaling pathways influencing cell survival and apoptosis [27]. Thus, GRP78 is a key regulator of the unfolded protein response, which is induced in tumorigenesis, but also upon the viral infection of cells [28]. Our data indicated that the over-expression of KDELR2 reduced the surface expression, but did not or only slightly reduced the total expression of the chaperons calnexin and GRP78 (Figure 5A,B). This also suggests that a higher concentration of KDELR2 leads to the retention of an increased fraction of calnexin and GRP78 in the ER. Thus, fewer chaperones are available to interact with MV-H, which consequently reduces the transport of MV-H to the cell surface. This view is supported by the clearly separated localization of MV-H and KDELR2 in infected cells (Figure 4A), suggesting that KDELR and MV-H compete to bind to chaperones and are sequestered from each other. In addition to the lack of surface transportation, MV-H in the absence of chaperones may be un- or misfolded and eventually be directed to a degradative pathway.

The shRNA-mediated silencing of KDELR2 in Vero-A3G cells demonstrated that the anti-MV activity of A3G is indeed mediated by this host factor (Figure 1B,C). The finding that KDELR2-specific shRNA leads to a complete compensation of the restrictive effect may be due to the fact that the silencing very efficiently reduces the expression of KDELR2 below the level of endogenous basic expression, which may enable an even better replication of MV than found in parental Vero cells. In summary, our data indicated that ectopic expression of A3G in Vero cells exerted its antiviral effect against MV by the induction of two host factors, REDD1 [2] and KDELR2.

In this paper, the role of KDELR2 was defined in the immortalized cell lines Vero and CEM-SS T, which provokes the question if this might be similar in primary cells. In primary cells, A3G is induced by cytokines such as interferons, interleukin-2, and interleukin-15, depending on the cell type [29,30,31,32,33,34]. Induction of A3G in primary human peripheral blood lymphocytes (PBL) by phytohemagglutinin (PHA), IL-2, and type I interferon also leads to increased KDELR2 expression (own observation, not shown). Due to the plethora of cytokine effects, the investigation of A3G-dependent consequences for host factor expression such as KDELR2 requires the ectopic expression of A3G and/or specific inhibition by interfering RNA. An attempt in primary human PBL recently revealed that A3G has the inherent capacity to regulate genes coding for STAT3, NF-κB, CCL5, IL-6, IL-4, IFN-γ, IL-10, and IL-17 as well as the ability to program T cell plasticity [35]. In addition, KDELR2 silencing may generally impair the homeostasis and functions of primary lymphocytes, as described recently for KDELR1 [23]. Further unbiased approaches in various primary cell types may be performed to capture all involved genes and to elucidate the role of KDELR2 for the replication of viruses.
